# SLC11A1 associated with tumor microenvironment is a potential biomarker of prognosis and immunotherapy efficacy for colorectal cancer

**DOI:** 10.3389/fphar.2022.984555

**Published:** 2022-11-09

**Authors:** Yiming Ma, Lei Zhan, Jun Yang, Jingdong Zhang

**Affiliations:** ^1^ Medical Oncology Department of Gastrointestinal Tumors, Cancer Hospital of China Medical University, Liaoning Cancer Hospital and Institute, Shenyang, China; ^2^ Medical Oncology Department of Breast Tumors, Cancer Hospital of China Medical University, Liaoning Cancer Hospital and Institute, Shenyang, China

**Keywords:** tumor microenvironment, CRC, immunotherapy, prognosis, SLC11A1

## Abstract

Colorectal cancer (CRC) is one of the most lethal cancers of the digestive system. The tumor microenvironment (TME) plays a central role in the initiation and development of CRC. However, little is known about the modulation mechanism of the TME in CRC. In our study, we attempted to identify a biomarker related to the TME modulation that could serve as a potential prognostic biomarker for CRC. We identified differentially expressed genes between the ImmuneScore high/low and StromalScore high/low groups. Using univariate COX regression analysis and hub gene analysis (cytoHubba), SLC11A1 was identified as the only candidate gene for subsequent analysis. CIBERSORT, EPIC, MCPcounter, and immunogenic cell death were performed to evaluate the effect of SLC11A1 on the TME. We also collected samples and performed Real-time quantitative PCR to verify the expression levels of SLC11A1 in CRC and adjacent normal tissues. The IMvigor210 cohort, TIDE score, and immunophenoscore (IPS) were used to analyze the association between SLC11A1 and immunotherapy efficacy. SLC11A1 was highly expressed in CRC tissues compared with its expression in normal colorectal tissues and was associated with poor prognosis and advanced clinicopathological stages. Gene set enrichment analysis showed that TGF-β pathways, JAK-STAT pathways, and angiogenesis were significantly enriched in the high-SLC11A1 group. Single-cell analysis validated the correlation between SLC11A1 and the TME. Using CIBERSORT, EPIC, and MCPcounter algorithms, we found that there was more macrophage and fibroblast infiltration in the SLC11A1 high-expression group. Meanwhile, high-SLC11A1 patients had lower IPS scores, higher TIDE scores, and fewer immunotherapy benefits than those of low-SLC11A1 patients. In conclusion, SLC11A1 plays a crucial role in the TME and could serve as a potential biomarker for poor prognosis and immunotherapy efficacy in CRC.

## Introduction

Colorectal cancer (CRC) is one of the most common gastrointestinal cancers worldwide, with high mortality rates (Siegel et al., 2018; Solano-Iturri et al., 2020; Sung et al., 2021). Currently, the incidence of CRC is increasing, and it is predicted that more than two million new cases will occur by the year 2030 (Arnold et al., 2017). Several factors related to carcinogenesis have been identified, including smoking, obesity, alcohol intake, and physical activity (Jayasekara et al., 2018). However, the precise molecular mechanisms underlying CRC development remain unclear. Colorectal adenocarcinoma is the most common type of CRC (Luo et al., 2019). CRC formation takes place through a multi‐stage process, from normal mucosa to adenoma, and finally to cancer tissue (Strum, 2016; Hauptman et al., 2018). Due to the lack of early indications and specific biomarkers, most CRC patients are diagnosed at advanced stages, which leads to poor prognosis. Although treatment methods for CRC have recently improved, the mortality rate remains high. Immune checkpoint inhibitors (ICIs) are novel antitumor drugs that have shown promising therapeutic efficacy in some types of cancers, such as melanoma, non-small-cell lung cancer, and urinary system cancers (Hamid et al., 2013; Sharma and Allison, 2015). However, the clinical benefits of immunotherapy vary widely among patients and conventional ICIs do not offer optimal clinical efficacy for most patients with advanced CRC (Morse et al., 2020). Potential indicators for predicting immunotherapy response, such as expression levels of immune checkpoints, tumor mutation burden (TMB), and neoantigens, are limited by tumor heterogeneity (Liu et al., 2022). Therefore, it is essential to develop a novel biomarker to predict the prognosis and immunotherapy benefits for patients with CRC.

The tumor microenvironment (TME), which is composed of tumor cells, immune cells, blood vessels, extracellular matrix, and fibroblasts, is considered to play a central role in tumorigenesis and tumor progression (Zhang et al., 2018; Schürch et al., 2020). TME may promote cancer progression by affecting immune surveillance and diminish the ability of chemotherapy to target tumor cells (Laplane et al., 2019). Recent research has shown that the initiation of CRC depends on the interaction between tumor cells and the TME (Ziegler et al., 2018). However, the interaction between CRC and the TME remains unknown. Therefore, it would be in the best interest of the medical field to further investigate the TME of CRC and identify factors related to TME modulation.

SLC11A1, also known as natural resistance-associated macrophage protein-1, is a member of the solute-carrier family. SLC11A1 was initially reported to fight several types of pathogens, and some studies have shown that SLC11A1 plays a role in innate immunity, autoimmune diseases, and infection (Stewart et al., 2010; Li et al., 2011; Neves et al., 2011; Cunrath and Bumann, 2019). In tuberculosis patients, the low expression and variation of SLC11A1 may impair immunologic response to tuberculosis (Shahzad et al., 2022). However, the roles of SLC11A1 in CRC have not been reported.

In the present study, we applied the ESTIMATE (Estimation of STromal and Immune cells in MAlignant Tumor tissues using Expression data) algorithm, hub gene analysis, and univariate COX regression and identified a TME-related factor in CRC, solute carrier family 11 member 1 (SLC11A1). The prognostic value of SLC11A1 has been evaluated in CRC and other digestive tract cancers. CIBERSORT, EPIC, MCPcounter, gene set enrichment analysis (GSEA), and single-cell analysis were used to further assess the potential effects of SLC11A1 in the TME and immunotherapy. Our findings provide a potential prognostic biomarker and may help in the individual selection of immunotherapy for patients with CRC.

## Materials and methods

### Raw data collection

The transcriptome profiles and corresponding clinical data on primary colon adenocarcinoma (COAD) and rectal adenocarcinoma (READ) were collected from TCGA database using TCGAbiolinks (Colaprico et al., 2016) (Supplementary Table S1). This study enrolled 612 patients with complete prognostic information. We also collected TCGA pan-cancer RNA-seq data in the TPM format processed by Toil from UCSC Xena (Vivian et al., 2017) (https://xenabrowser.net/datapages). Microarray data from GSE17536, including 177 patients, were downloaded from the GEO database (Smith et al., 2010). Moreover, we also downloaded data from the immunotherapy-related dataset (IMvigor210) using IMvigor210CoreBiologies (Mariathasan et al., 2018). In the IMvigor210 cohort, 298 advanced urothelial cancer patients with complete clinical data were included in our study.

### Identification of differentially expressed genes related with tumor microenvironment

A previous study has shown the application of ESTIMATE algorithm to microarray and RNA-sequencing data might help to clarify the role of the TME and provide novel insights into genomic alterations (Yoshihara et al., 2013). ESTIMATE algorithm is an efficacy method to screen TME-related genes (Cheng et al., 2021; Guo et al., 2021; Wu et al., 2021). In our study, ImmuneScore, StromalScore, and ESTIMATEScore were calculated using the ESTIMATE R package, based on the transcriptome data of TCGA-CRC patients. These scores were assessed in patients at different pathological stages: T stage, N stage, and M stage.

We used the DEseq2 R package to analyze differentially expressed genes (DEGs) between patients with high and low ImmuneScores to identify genes associated with immune cells (Love et al., 2014). Moreover, we used the DEseq2 R package to analyze DEGs between patients with high and low StromalScores to identify genes associated with stromal cells. The DEGs were defined as genes with |Log2FC| > 1.5 and adjusted *p* < 0.01. The overlapping DEGs in the StromalScore and ImmuneScore groups were considered TME-related genes. Kyoto Encyclopedia of Genes and Genomes (KEGG) and Gene Ontology (GO) enrichment analyses were performed using the DEGs associated with TME using the R package clusterProfiler (Yu et al., 2012). The enrichment terms were considered significant with a *p*-value < 0.05.

### CytoHubba and univariate COX regression analyses

We used the STRING database to construct a protein–protein interaction (PPI) network (confidence score > 0.9) based on the common DEGs in both the StromalScore and ImmuneScore groups (Szklarczyk et al., 2019). To identify hub genes, we used the cytoHubba plugin and acquired the top 30 hub genes using the multi-network clustering (MNC) algorithm (Chin et al., 2014). Univariate COX regression analysis was performed to screen for DEGs associated with overall survival (OS) time. Genes with *p* < 0.05 were shown in the plot.

### Gene set enrichment analysis

The expression differences between low-SLC11A1 and high-SLC11A1 groups were identified using DESeq2. To identify key pathways and biological processes associated with SLC11A1, GSEA was performed using KEGG and Hallmark gene sets as target sets.

### Tumor microenvironment-related analyses of SLC11A1

The Immuno-Oncology-Biological-Research (IOBR) R package integrates common algorithms for estimating the TME cells (Zeng et al., 2021). The TME of CRC samples was estimated using three different algorithms, including the CIBERSORT, EPIC, and MCPcounter algorithms, using the IOBR R package. We extracted immunogenic cell death (ICD)-related genes from a previous study and found differences in the expression of ICD-related genes between low-SLC11A1 and high-SLC11A1 groups (Garg et al., 2016). To further investigate SLC11A1 expression in the TME of CRC, we used the Tumor Immune Single Cell Hub database, which contains single-cell transcriptome profiles of 27 types of cancer (Sun et al., 2021). The cell location of SLC11A1 was determined using single-cell data in GSE146771.

### Evaluation of SLC11A1 expression and immunotherapy response

A previous study on LUAD has shown that TIDE and IPS scores are valid scoring schemes to predict immunotherapy response. TIDE is one of the most effective methods for assessing the immune escape of tumors by analyzing their expression profiles (Jiang et al., 2018). Higher TIDE scores indicate that tumor cells are more likely to escape from immune surveillance, which means lower immunotherapy efficacy. Therefore, we calculated the TIDE scores based on RNA-seq of TCGA-CRC to analyze the relationship between SLC11A1 expression and immunotherapy response. The immunophenoscore (IPS) is also defined as a crucial factor in predicting the efficacy of ICIs (Charoentong et al., 2017). Patients with a higher IPS indicated a higher efficacy of ICIs. The IPS data of TCGA-CRC patients were downloaded from the TCIA database (https://tcia.at/home) to identify the relationship between IPS and SLC11A1 expression.

### Real-time quantitative PCR

We collected 15 paired CRC tissues and adjacent normal colorectal tissues from the Liaoning Cancer Hospital. Total RNA was extracted from the tissue specimens using TRIzol reagent. Total RNA was treated with kits from Takara (Shiga, Japan) to remove genomic DNA and to conduct reverse transcription. Real-time quantitative PCR (RT-qPCR) was performed using TB GREEN Premix Ex Taq (Takara). The primer sequences were: *GAPDH*-F, 5′ GGA​AGC​TTG​TCA​TCA​ATG​GAA​ATC 3′; *GAPDH*-R 5′ TGA​TGA​CCC​TTT​TGG​CTC​CC 3′; *SLC11A1*-F 5′ GTC​CGT​CTC​CTT​TAT​CAT​CAA​CCT 3′; *SLC11A1*-R 5′ GAA​GCC​CTC​CAT​CAC​GAA​CTG 3′.

### Statistical analyses

All statistical analyses were conducted using R software (version 4.1.0). Kaplan–Meier (KM) curves were constructed to evaluate the relationship between OS rates and SLC11A1 expression. The survminer R package was used to identify the optimum cutoff point of SLC11A1 expression to classify CRC patients into low-SLC11A1 and high-SLC11A1 groups. The Wilcoxon test was used to compare differences between the two groups. The Kruskal–Wallis test was used to compare the differences between multiple groups. Univariate COX regression was performed to screen the prognostic factors of CRC using the survival R package. Statistical significance was set to *p*-value < 0.05.

## Results

### Relationship of ImmuneScore, StromalScore, and ESTIMATEScore with clinicopathological features

**FIGURE 1:**
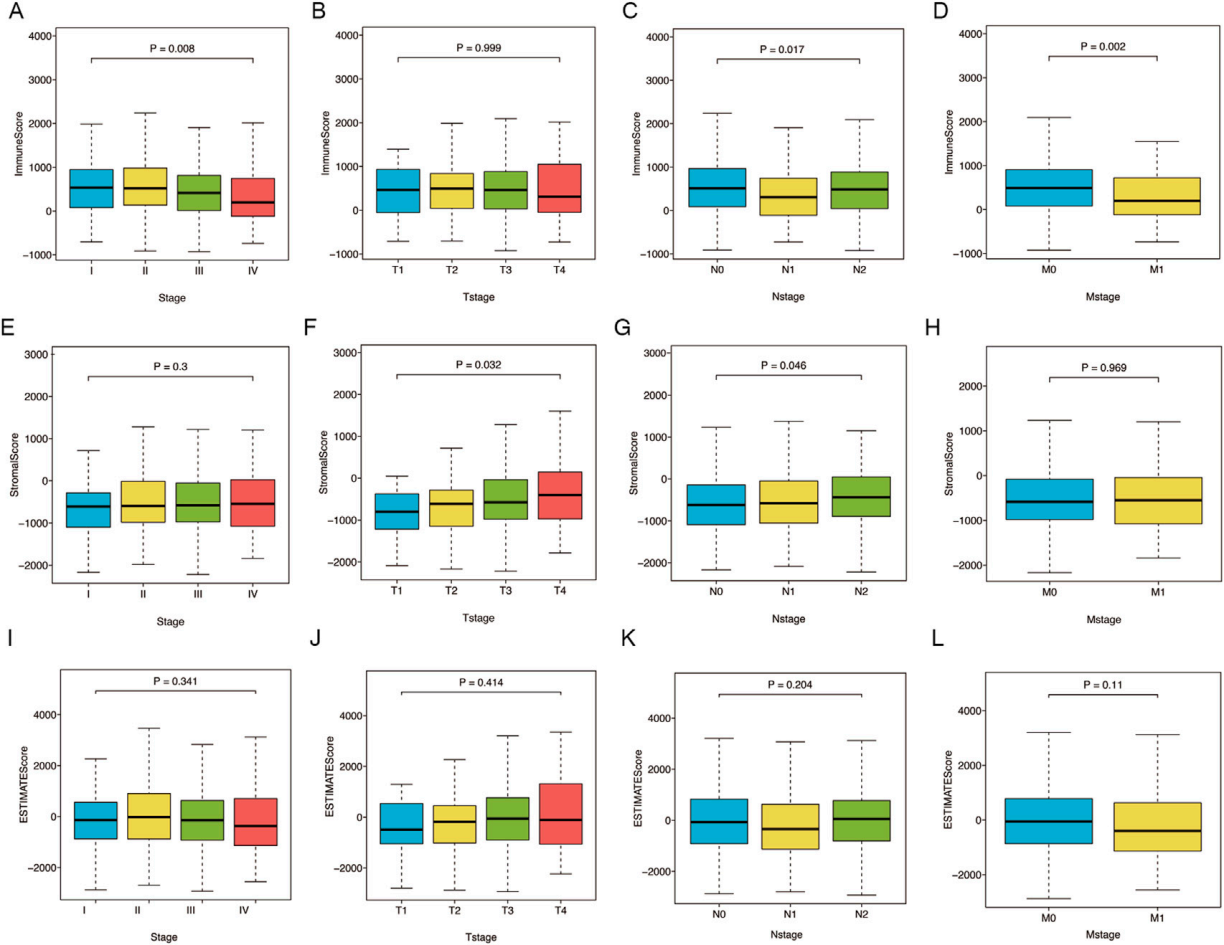
Relations of ImmuneScore, StromalScore, and ESTIMATEScore with clinicopathological stages. **(A–D)** Relations of ImmuneScore with pathological stage (*p* = 0.008), T stage (*p* = 0.999), N stage (*p* = 0.017) and M stage (*p* = 0.002). **(E–H)** Relations of StromalScore with pathological stage (*p* = 0.3), T stage (*p* = 0.032), N stage (*p* = 0.046) and M stage (*p* = 0.969). **(I–L)** Relations of ESTIMATEScore with pathological stage (*p* = 0.341), T stage (*p* = 0.414), N stage (*p* = 0.204) and M stage (*p* = 0.11). Statistical difference of four groups was compared by the Kruskal–Wallis test.

First, we analyzed the ImmuneScore, StromalScore, and ESTIMATEScore of TCGA-CRC patients with different clinicopathological characteristics (Figure 1). The ImmuneScore was significantly associated with pathological stage (*p* = 0.008), N stage (*p* = 0.017), and M stage (*p* = 0.002) (Figures 1A,C,D). Additionally, the StromalScore increased significantly in patients with advanced T stage (*p* = 0.032) and N stage (*p* = 0.046) (Figures 1F,G). However, ESTIMATEScore was not significantly associated with pathological stage, T stage, N stage, or M stage (Figures 1I–L). These results indicate that the immune and stromal components of CRC tissues may affect tumor proliferation and metastasis.

### Identification of genes related with tumor microenvironment

**FIGURE 2:**
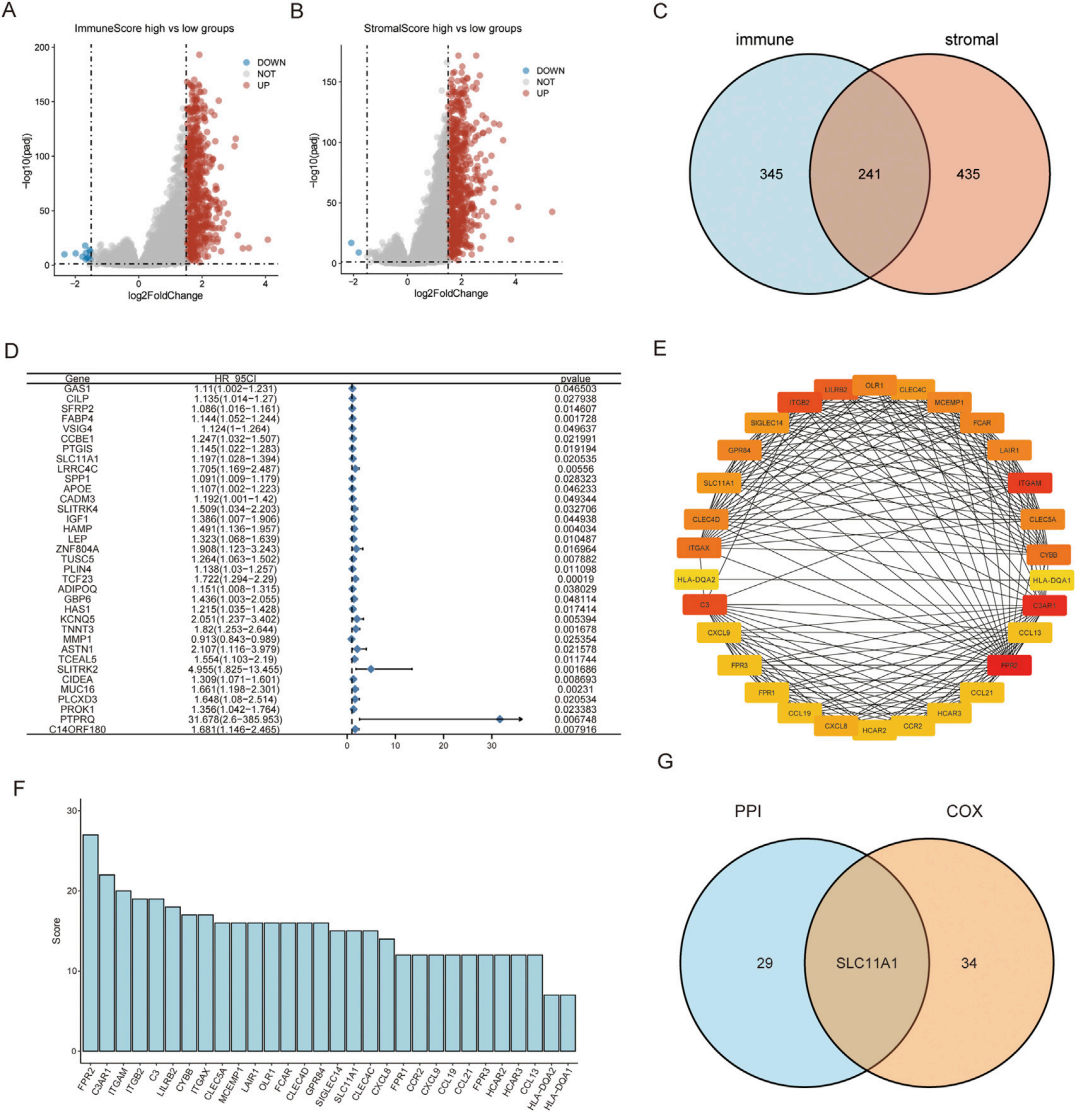
SLC11A1 was identified as the hub gene for TME. **(A,B)** Volcano plots for DEGs identified by comparison of high-ImmuneScore vs. low-ImmuneScore groups and high-StromalScore vs. low-StromalScore groups. **(C)** Venn plot showed 241 common factors with the DEGs from ImmuneScore and StromalScore analyses. **(D)** Thirty-five factors with *p*-value < 0.05 by univariate Cox analysis. **(E)** Thirty genes with the screened with MNC algorithm. **(F)** The bar chart showed MNC scores of the 30 genes. **(G)** SLC11A1 was identified in the intersection of the two modules.

A comparison between the high/low ImmuneScore and StromalScore groups was performed to identify genes related to the TME. In total, there were 586 DEGs between the high/low-ImmuneScore groups and 676 DEGs between the high- and low-StromalScore groups (Figures 2A,B). In total, there were 241 DEGs in both the ImmuneScore and StromalScore groups (Figure 2C **)**, and 241 DEGs were considered TME-related genes. In addition, we analyzed the related functions and pathways of the genes using GO and KEGG enrichment analyses. The genes were mainly enriched in immune-related GO terms, such as chemokine activity, complement C3b binding, immunoglobulin binding, immune receptor activity, regulation of T cell adhesion, and leukocyte cell-cell adhesion (Supplementary Figure 1A). KEGG enrichment analysis indicated that the enriched genes were related to immune pathways, such as neutrophil extracellular trap formation, cytokine-cytokine receptor interaction, chemokines, IL-17, and antigen processing and presentation pathways Supplementary Figure 1B). The results of the enrichment analysis illustrated that these genes seemed to be associated with immune-related functions and might play predominant roles in the TME (Supplementary Tables S2, S3).

We conducted a univariate COX regression analysis to identify the prognostic factors of CRC among the 241 TME-related genes. Then, 35 significant genes were acquired and are shown in the forest plot (*p* < 0.05) **(** Figure 2D). We identified the top 30 hub genes amongst these using the MNC algorithm and identified the hub genes ranked by the MNC score (Figures 2E,F). We performed an intersection analysis between the top 30 hub genes in the PPI network and 35 significant genes associated with prognosis using univariate COX regression. SLC11A1 was the only gene that overlapped between the two groups (Figure 2G).

### Survival and clinical features analyses of SLC11A1

**FIGURE 3:**
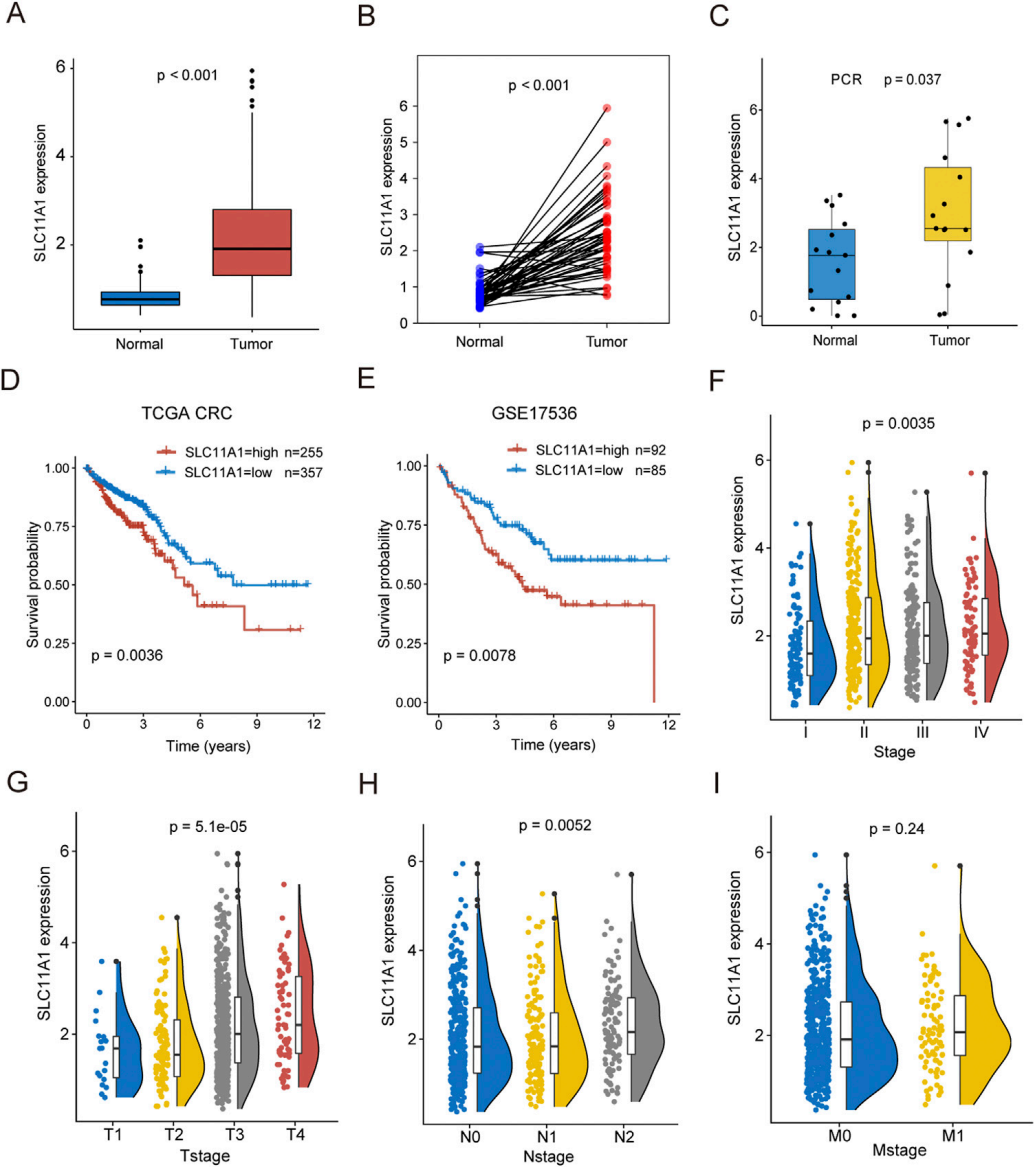
High expression of SLC11A1 indicated poor prognosis of CRC patients. **(A)** Expression differences of SLC11A1 between CRC and adjacent normal samples. **(B)** Expression differences of SLC11A1 between paired CRC and adjacent normal samples. **(C)** RTq-PCR demonstrated that the high expression of SLC11A1 in CRC. **(D,E)** KM curves showed different outcomes of overall survival between high- and low-SLC11A1 groups by using TCGA-CRC and GSE17536. **(F–I)** The associations between SLC11A1 and clinicopathological stages (Stage, T stage, N stage and M stage). ns, *p* ≥ 0.05; *, *p* < 0.05; **p < 0.01; ***p < 0.001. Statistical difference of two groups was compared by the Wilcoxon test and statistical difference of four groups was compared by the Kruskal–Wallis test.

SLC11A1 expression was higher in CRC tissues than that in normal tissues (Figures 3A,B). Similarly, the RT-qPCR results also validated that SLC11A1 expression was upregulated in tumor tissues (Figure 3C). According to the KM plots from TCGA and GSE17536, the high-SLC11A1 group had a worse prognosis than the low-SLC11A1 group (*p* = 0.0036 and *p* = 0.0078, respectively) (Figures 3D,E). We also evaluated the relationship between SLC11A1 mRNA levels and clinicopathological stages. Higher SLC11A1 expression was observed in the progression of pathological stage, T stage, and N stage (Figures 3F–H). There was no significant difference between SLC11A1 expression and the M stage (Figure 3I). These results suggest that CRC with high SLC11A1 mRNA expression is closely related to advanced clinicopathological cancer stages and poor prognosis.

We also investigated the prognostic value of SLC11A1 using pan-cancer data from TCGA. In other digestive tract cancers, such as STAD, PAAD, and LIHC, high expression of SLC11A1 was also associated with poor prognosis (Supplementary Figures S2A–C). Similar results were found in KIRC, BLCA, LAML, HNSC, UCEC, and LGG/GBM patients (Supplementary Figures S2D–J).

### Mutation landscapes between high-SLC11A1 and low-SLC11A1 groups

**FIGURE 4:**
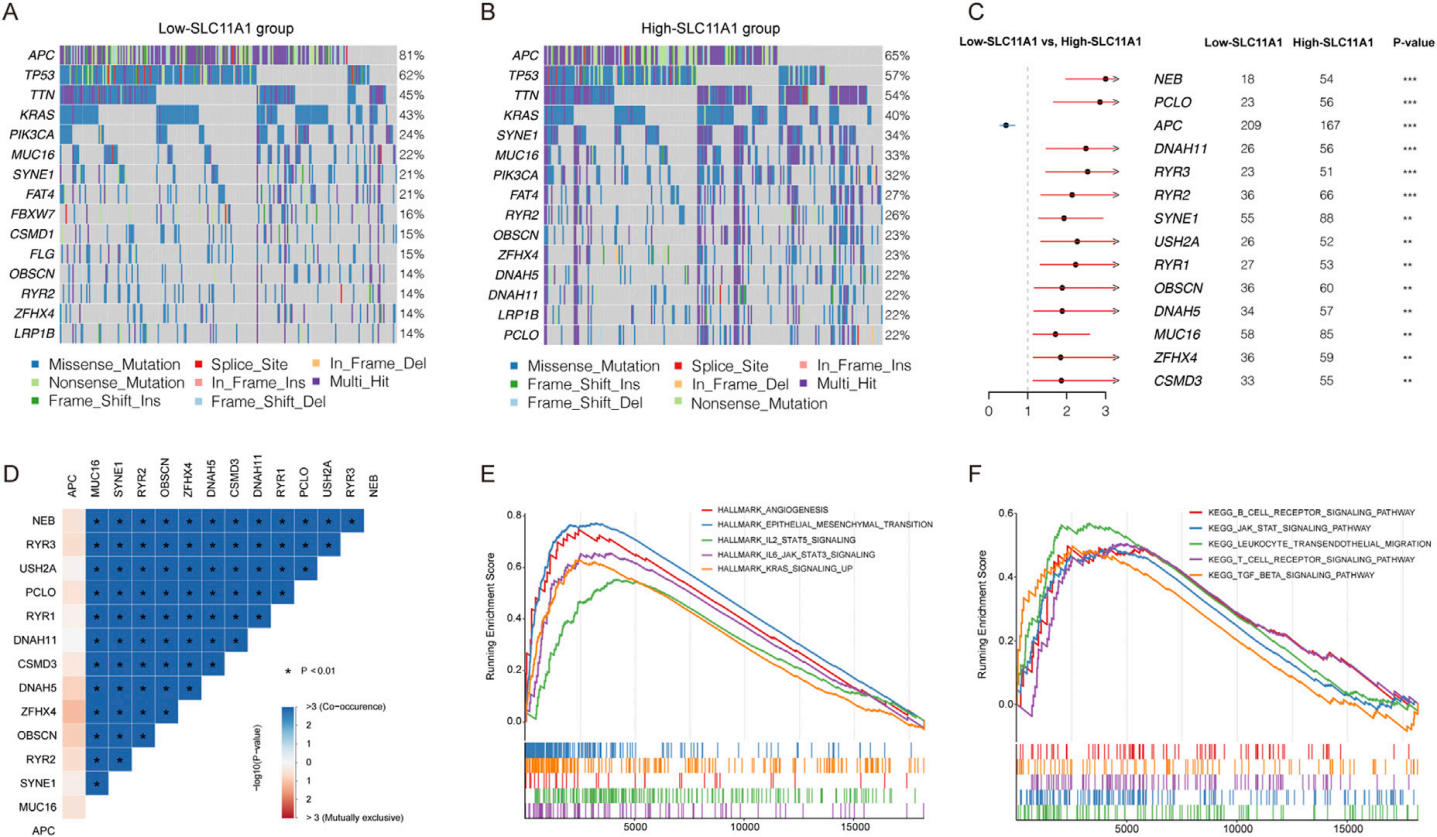
SLC11A1 expression was related with CRC mutation status. **(A,B)** Top 15 genes with the highest mutation frequency in the low- and high-SLC11A1 groups. **(C)** The forest plot illustrated the differently mutational genes between the two groups (*p* < 0.01) **(D)** Mutation correlations of differently mutational genes. **(E,F)** GSEA of SLC11A1.

To explore the potential mechanism between low-SLC11A1 and high-SLC11A1 groups, we analyzed the mutation profiles of the two groups. Fifteen genes with the highest mutational frequencies were observed in waterfall plots (Figures 4A,B). Among the mutated genes, TP53 and KRAS were mostly observed to have missense mutations. We then identified the differentially mutated genes using Fisher’s exact test (*p* < 0.01). APC showed a higher mutational frequency in the low-SLC11A1 group, and NER, PCI, DNAH11, RYR3, RYR2, SYME1, USH2A, RYR1, OBSCN, DNAH5, MUC16, ZFHX4, and CSM3 showed a higher mutational frequency in the high-SLC11A1 group (Figure 4C). Mutational correlations were also analyzed with the differentially mutated genes, which revealed that all genes except APC showed mutational co-occurrence with each other (Figure 4D).

### Correlation between SLC11A1 expression and immune cell infiltration

To identify key pathways and biological functions associated with SLC11A1, GSEA was performed in the high/low SLC11A1 groups. Angiogenesis, epithelial-mesenchymal transition, and JAK-STAT were enriched in the high-SLC11A1 group in the Hallmark collection (Figure 4E). KEGG pathways such as the B cell receptor, JAK-STAT, and T cell receptor pathways were enriched in the high-SLC11A1 group (Figure 4F). GSEA results indicated that SLC11A1 might play a crucial role in the TME of CRC.

**FIGURE 5:**
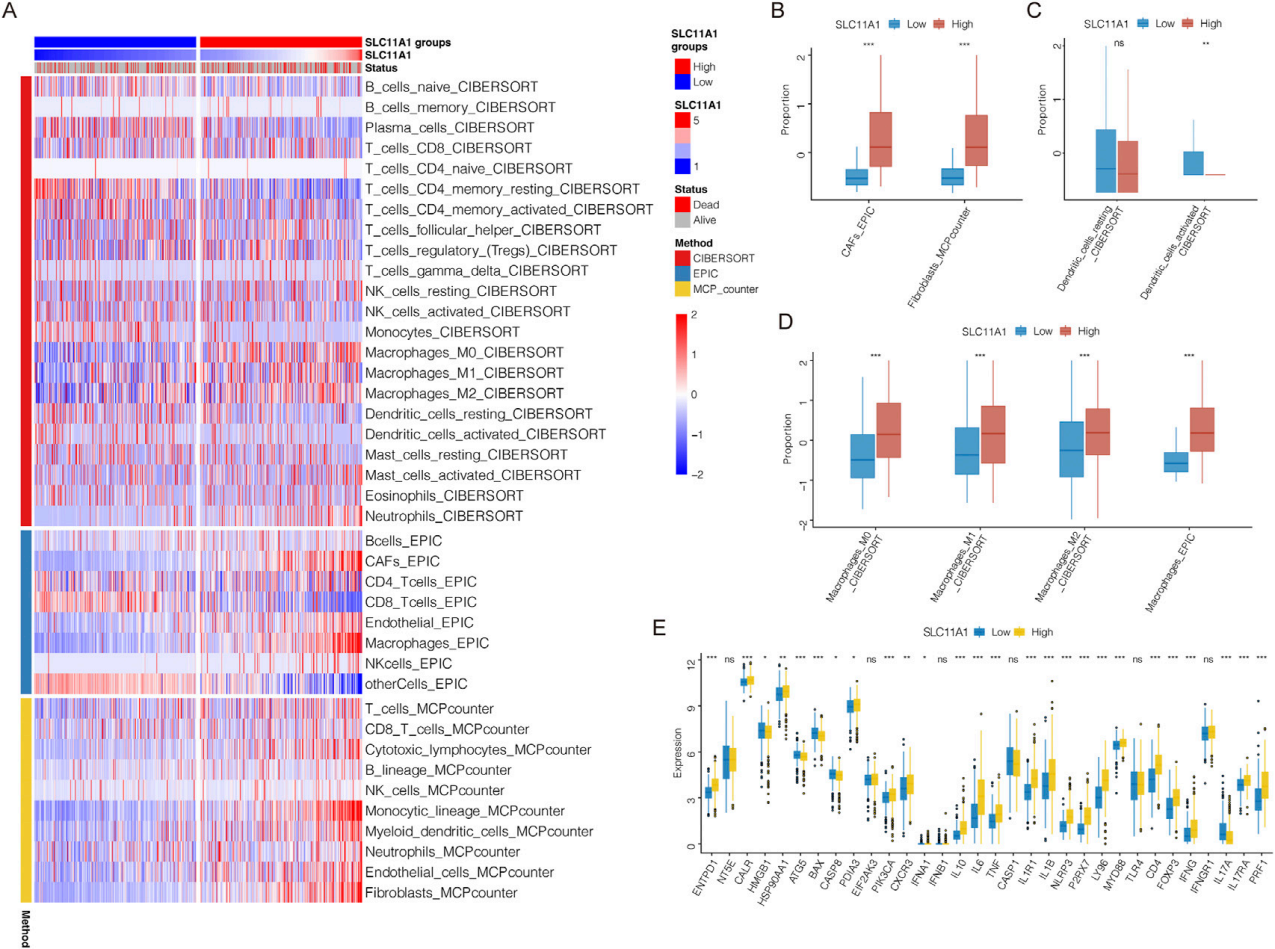
TME landscape in the low/high-SLC11A1 groups. **(A)** The heatmap showed the infiltration of immune and stromal cells. **(B–D)** Difference analyses of infiltration levels of CAFs, fibroblasts, dendritic cells and macrophages between the low/high-SLC11A1 groups. **(E)** Expression differences of ICD-related genes between the low/high-SLC11A1 groups. Statistical difference was compared by the Wilcoxon test.

**FIGURE 6:**
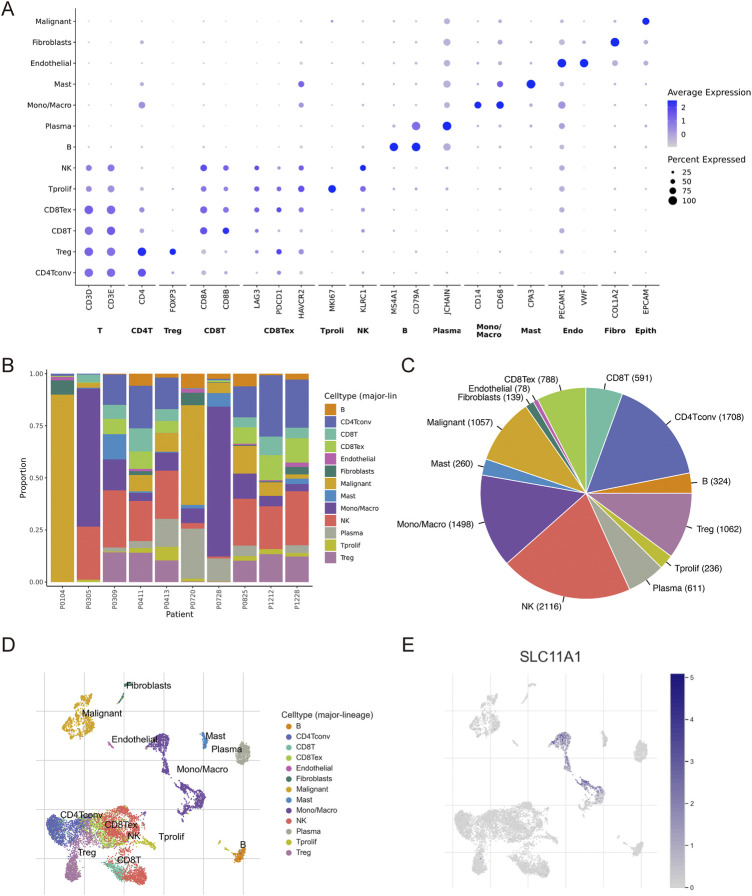
Single-cell analysis to explore the cell location of SLC11A1. **(A)** The bubble chart showed the expression markers of cells in TME with GSE146771. **(B,C)** Proportion of 12 types of TME-related cells. **(D,E)** SLC11A1 expression was mainly distributed in monocytes and macrophages.

Immune and stromal cells of CRC samples were estimated using CIBERSORT, EPIC, and MCPcounter (Figure 5A). Our results indicated that fibroblasts, cancer-associated fibroblasts (CAFs), and macrophages (M0, M1, and M2) infiltrated more in the high-SLC11A1 group (Figures 5B,D). However, the proportion of activated dendritic cells was higher in the low-SLC11A1 group (Figure 5C). We also analyzed ICD-related gene expression in the two groups and found that 26 genes showed significant differences (Figure 5E). IL-10, IL-6 (immunosuppressive cytokines), and FOXP3 (a significant marker of Treg cells) were expressed at higher levels in the high-SLC11A1 group. Using single-cell transcriptomic analysis, we investigated the localization of SLC11A1 in CRC cells. The expression of cell markers in the TME is shown in the bubble chart (Figure 6A). The distribution and proportion of the 12 types of TME-related cells in samples from GSE146771 are shown in Figures 6B,C. In this dataset, SLC11A1 expression was mainly distributed in monocytes and macrophages (Figures 6D,E). These results demonstrated that SLC11A1, mainly expressed in macrophages and monocytes, might have an immunosuppressive effect in the CRC TME.

### Immunotherapy-related analyses

**FIGURE 7:**
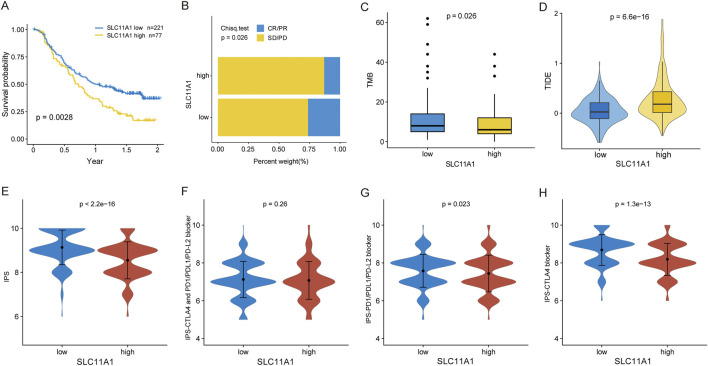
High SLC11A1 expression was related with immunotherapy resistance. **(A)** KM plot for the low- and high-SLC11A1 groups in IMvigor210 cohort. **(B)** Bar plot showed immunotherapy efficacy of the two groups. **(C)** Box plot showed the TMB difference of the two groups by the Wilcoxon test. **(D)** TIDE scores of TCGA-CRC patients with higher SLC11A1 and lower SLC11A1 expression by the Wilcoxon test. **(E–H)** Evaluation of IPS differences between the low- and high-SLC11A1 groups with TCIA database by the Wilcoxon test.

We examined the relationship between SLC11A1 expression and immunotherapy response. In the immunotherapeutic IMvigor210 cohort, patients with higher *SLC11A1* expression had longer OS (*p* = 0.0028) (Figure 7A). Similarly, the high-SLC11A1 group yielded a lower response rate than the low-SLC11A1 group (Figure 7B). Patients with high SLC11A1 expression had lower TMB than that of patients with low SLC11A1 expression (Figure 7C). We evaluated the TIDE scores of TCGA-CRC samples to predict the immunotherapy response of the patients. The high-SLC11A1 group had higher TIDE scores, which indicated that high-SLC11A1 patients with enhanced immune evasion might have had a poor response to immunotherapy (Figure 7D). Moreover, we evaluated the association between SLC11A1 and the IPS (Figures 7E–H). IPS, IPS-PD1/PD-L1/PD-L2, and IPS-CTLA4 levels were significantly increased in the SLC11A1-low group. These findings demonstrate that SLC11A1 might mediate cancer immune escape and inhibit the sensitivity to immunotherapy.

## Discussion

In this study, we screened TME-related prognostic biomarkers for CRC patients. Through multiple bioinformatics analysis methods, we demonstrated that SLC11A1 could serve as a significant indicator in the TME for predicting prognosis and immunotherapy resistance in CRC. First, we calculated the ImmuneScore and StromalScore of TCGA-CRC patients using the ESTIMATE algorithm and found that the ImmuneScore and StromalScore were closely related to clinicopathological stages. To identify genes associated with TME, DEGs were identified between the ImmuneScore high/low groups and the StromalScore high/low groups. In total, 241 DEGs associated with TME were identified, and SLC11A1 was identified as a hub prognostic gene.

We demonstrated that SLC11A1 was highly expressed in CRC tissues compared to that in normal tissues, and high-SLC11A1 patients had poor prognosis in CRC as well as other digestive cancers. Meanwhile, higher expression of SLC11A1 was associated with more fibroblast, CAF, and macrophage infiltration and less activated dendritic cell infiltration. Finally, our findings also revealed that increased SLC11A1 levels were correlated with decreased immunotherapy efficacy and poor prognosis in patients treated with immunotherapy. SLC11A1 was identified as a metabolism-related gene involved in predicting the prognosis of hepatocellular cancer (Zhu et al., 2021). Mutations in SLC11A1 could help estimate PD-L1 expression and predict responses to anti-PD-1 therapy in patients with gastric cancer (Menyhárt et al., 2018). At present, little is known about the function of SLC11A1 in CRC. In our study, we found that SLC11A1 was highly expressed in CRC tissues compared to that in normal colorectal tissues in TCGA, and similar results were demonstrated by RT-qPCR. Upregulated SLC11A1 expression in CRC is associated with poor prognosis and advanced clinicopathological stages (pathological, T, and N stages), which indicates that SLC11A1 tends to be an unfavorable factor in CRC patients. We also demonstrated that SLC11A1 is associated with poor prognosis in other common cancers of the digestive system, such as STAD, PAAD, and LIHC. The potential implications of SLC11A1 in the prognosis of patients with digestive tract cancers warrant further investigation.

In the TME, immune cell, blood vessel, and stromal components play crucial roles in the carcinogenesis and progression of cancer (Yao et al., 2020). Angiogenesis plays a key role in the proliferation and metastasis of primary CRC, and increased angiogenesis is associated with poor prognosis and recurrence (Liu et al., 2013; Kantola et al., 2014). Changes in immune function are among the most significant causes of CRC initiation. The tumor immune microenvironment is defined as the density, type, and location of tumor-infiltrating immune cells, which have a great influence on the development of CRC (Amicarella et al., 2017). An increasing number of studies have recognized the specific functions of macrophages and CAFs in CRC development (Peng et al., 2022; Yi et al., 2022). Activated fibroblast-derived CAFs are the most abundant cell type in the TME. CAFs, a popular topic of oncological research, have been reported to be involved in tumor progression. Increasing evidence has indicated that CAFs are related to resistance to chemotherapy, targeted treatment, and immunotherapy, and specific treatment for CAFs is expected to be an important adjunct of immunotherapy (Marusyk et al., 2016). In our study, we identified SLC11A1 as the key TME-related biomarker using the ESTIMATE algorithm, and the GSEA results further illustrated that SLC11A1 was correlated with angiogenesis and the JAK-STAT and TGF-β pathways. Higher SLC11A1 expression was associated with increased infiltration of CAFs and fibroblasts and less infiltration of activated dendritic cells. TGF-β has been proposed to induce activation of CAFs, which promotes the proliferation and metastasis of tumors (Chandra Jena et al., 2021). Therefore, we inferred that SLC11A1 could activate the TGF-β pathway to enhance the infiltration of CAFs in CRC. Myeloid dendritic cells are innate immune cells derived from bone marrow with the function of linking and activating adaptive immunity (Collin and Bigley, 2018). Both CD4 T and dendritic cells play significant roles in anti-tumor immunity.

In a data analysis of thyroid cancer, SLC11A1 is associated with macrophages and participates in the construction of a risk model for evaluating the prognosis of thyroid cancer patients (Zhuang et al., 2020). SLC11A1 modulates iron metabolism in macrophages and plays a crucial role in the activation of early-stage macrophages (Wyllie et al., 2002). A previous study also showed that SLC11A1 is expressed in macrophages in mice (Xu et al., 2005). In our study, SLC11A1 expression was related to high infiltration of macrophages (M0, M1, and M2) in the CRC TME. Furthermore, we also elucidated SLC11A1 expression in the TME by single-cell transcriptomic analysis. SLC11A1 is primarily expressed in monocytes and macrophages in the CRC TME. These results are consistent with those form previous studies. Macrophages play an important role in tumorigenesis and progression. In CRC, TME-related stimulation that induces the polarization of macrophages can modulate the growth and metastasis of tumor cells (Zhang et al., 2020). M1 macrophages overexpressing CD80 and CD86 are considered antineoplastic macrophages with immune promotion functions in the TME (Yunna et al., 2020). M2 macrophages, exhibiting an anti-inflammatory phenotype, play immunosuppressive roles and promote tumor progression (Murray et al., 2014). M1 and M2 macrophages show distinct phenotypes for tumor immunity and the polarization of macrophages from M2 to M1 phenotype could improve the immunotherapy effect (Xia et al., 2020). Therefore, based on all the results of the TME, we preliminarily considered that SLC11A1 regulates the TME in many ways, resulting in immunosuppression and the progression of CRC. Further experimental studies are warranted to demonstrate the relationship between SLC11A1 and macrophage in CRC and investigate how SLC11A1 impacts the efficacy of immunotherapy.

In various cancer types, immunosuppressive TME can inhibit the activity and anti-tumor ability of immunocytes (Mahata et al., 2020). Immune checkpoints expressed in tumor cells are associated with immune evasion and inhibition of anti-tumor immunity (Liu et al., 2020). In the CRC microenvironment, the expression levels of immune checkpoints in tumor cells can suppress the anti-tumor immunity of T cells (Masugi et al., 2017; Zhou et al., 2018). Nowadays ICD, as a novel type of regulated cell death, has been reported as an adjuvant strategy for ICIs (Rizvi and Gores, 2017; Irvine and Dane, 2020). Among ICD-related genes, IL-6, IL-10, and FOXP3 are significant markers associated with the suppression of the immune response (Harshyne et al., 2016; Layman et al., 2017; Smith et al., 2020). Our findings also showed that SLC11A1 was related to 26 ICD-related genes, and high-SLC11A1 patients had higher expression of IL-6, IL-10, and FOXP3 than that in low-SLC11A1 patients. Moreover, through three different methods, including the TIDE, IPS, and IMvigor210 datasets, we obtained consistent results. We found that high SLC11A1 expression was associated with immunotherapy resistance. Thus, these consistent results further demonstrate that SLC11A1 plays a crucial role in the suppression of anti-tumor immunity and is a potential therapeutic target for CRC.

Here, we propose SLC11A1 as a potential biomarker for prognosis and immunotherapy efficacy in patients with CRC. However, this study had some limitations. Independent CRC patients treated with immunotherapy are required, to evaluate the accuracy of SLC11A1 in predicting immunotherapy response. Although RT-qPCR was performed to examine the higher expression of SLC11A1 in CRC, other experimental methods are required to explore the specific functions of SLC11A1 *in vitro* or *in vitro*. Our research group is currently collecting more clinical samples from CRC patients. In future study, immunohistochemistry would be performed to further evaluate the expression levels and clinical values of SLC11A1 in CRC. We would use immunostaining to validate the relationship between SLC11A1 and immune cells, such as fibroblasts, cancer-associated fibroblasts (CAFs) and macrophages (M1 and M2). Further experimental studies are planned to explore roles of SLC11A1 in modulating CRC TME.

In conclusion, we revealed that SLC11A1 was associated with poor prognosis and immunotherapy resistance in CRC for the first time. SLC11A1 may be a potential biomarker for predicting prognosis and immunotherapy efficacy in CRC.

## Data Availability

The datasets presented in this study can be found in online repositories. The names of the repository/repositories and accession number(s) can be found in the article/Supplementary Material.

## Supplementary material

SUPPLEMENTARY FIGURE S1 GO **(A)** and KEGG **(B)** enrichment analyses for the overlapping DEGs of ImmuneScore and StromalScore groups.

SUPPLEMENTARY FIGURE S2 Prognostic value of SLC11A1 for other digestive system tumors. **(A–C)** In STAD, PAAD and LIHC, high expression of SLC11A1 was associated with poor outcomes of OS. **(D–I)** In KIRC, BLCA, LAML, HNSC, UCEC and LGG/GBM patients, high-SLC11A1 groups had lower survival rates than low-SLC11A1 groups.
