# Supplementary figures and images for: SLC11A1 associated with tumor microenvironment is a potential biomarker of prognosis and immunotherapy efficacy for colorectal cancer
Source: Front Pharmacol. 2022 Nov 9;13:984555. doi: 10.3389/fphar.2022.984555 (PMC9681808; doi:10.3389/fphar.2022.984555)

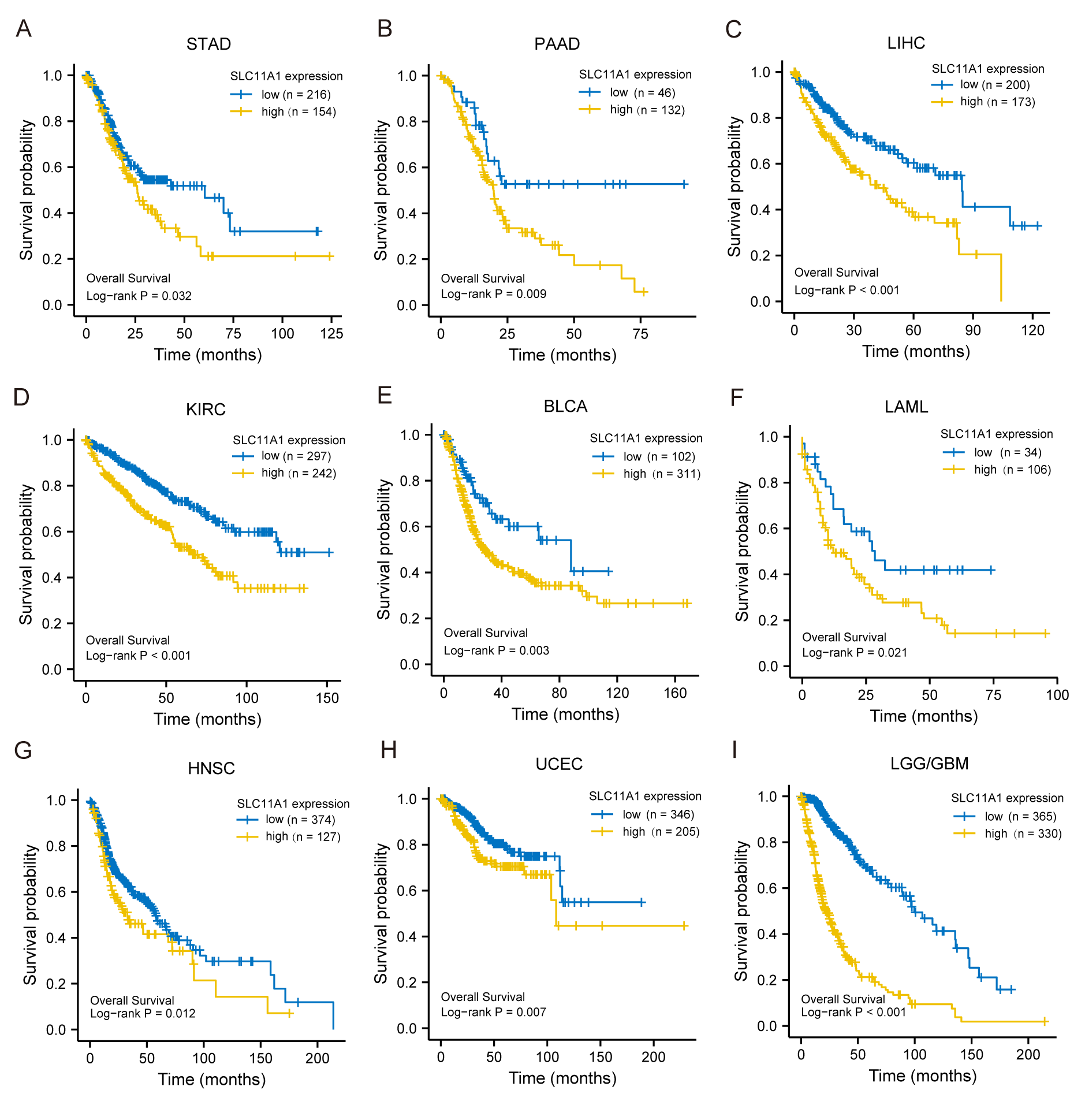

Supplement: Supplementary file 1 [file Image2.TIF]

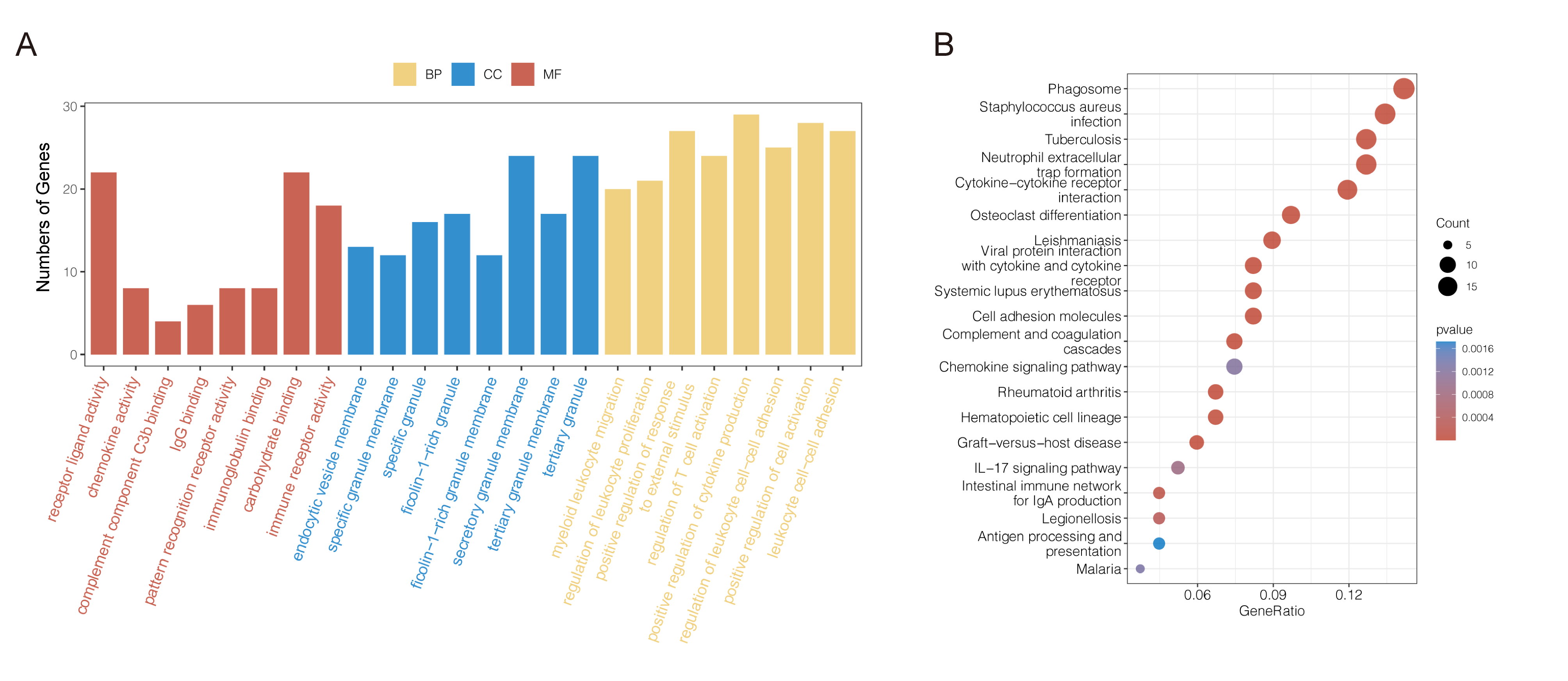

Supplement: Supplementary file 2 [file Image1.TIF]
