# Supplementary material for: SLC11A1 associated with tumor microenvironment is a potential biomarker of prognosis and immunotherapy efficacy for colorectal cancer
Source: Front Pharmacol. 2022 Nov 9;13:984555. doi: 10.3389/fphar.2022.984555 (PMC9681808; doi:10.3389/fphar.2022.984555)
Supplement: Supplementary file 3 [file Table2.DOCX]

**
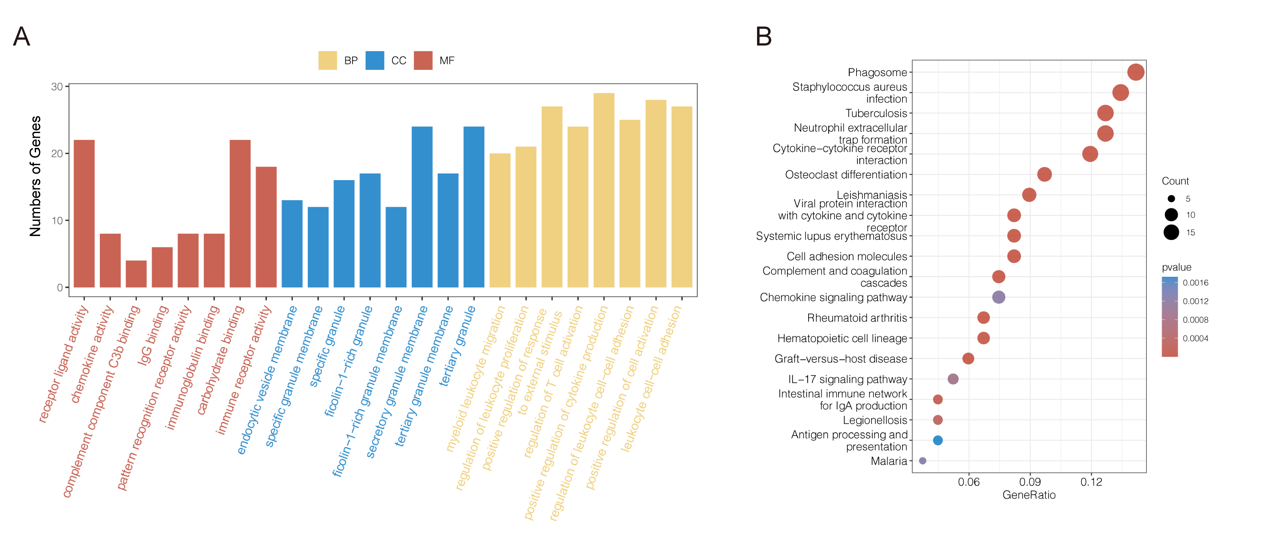
**

**Supplementary Figure 1** | GO (A) and KEGG (B) enrichment analyses for the overlapping DEGs of ImmuneScore and StromalScore groups.

.

**
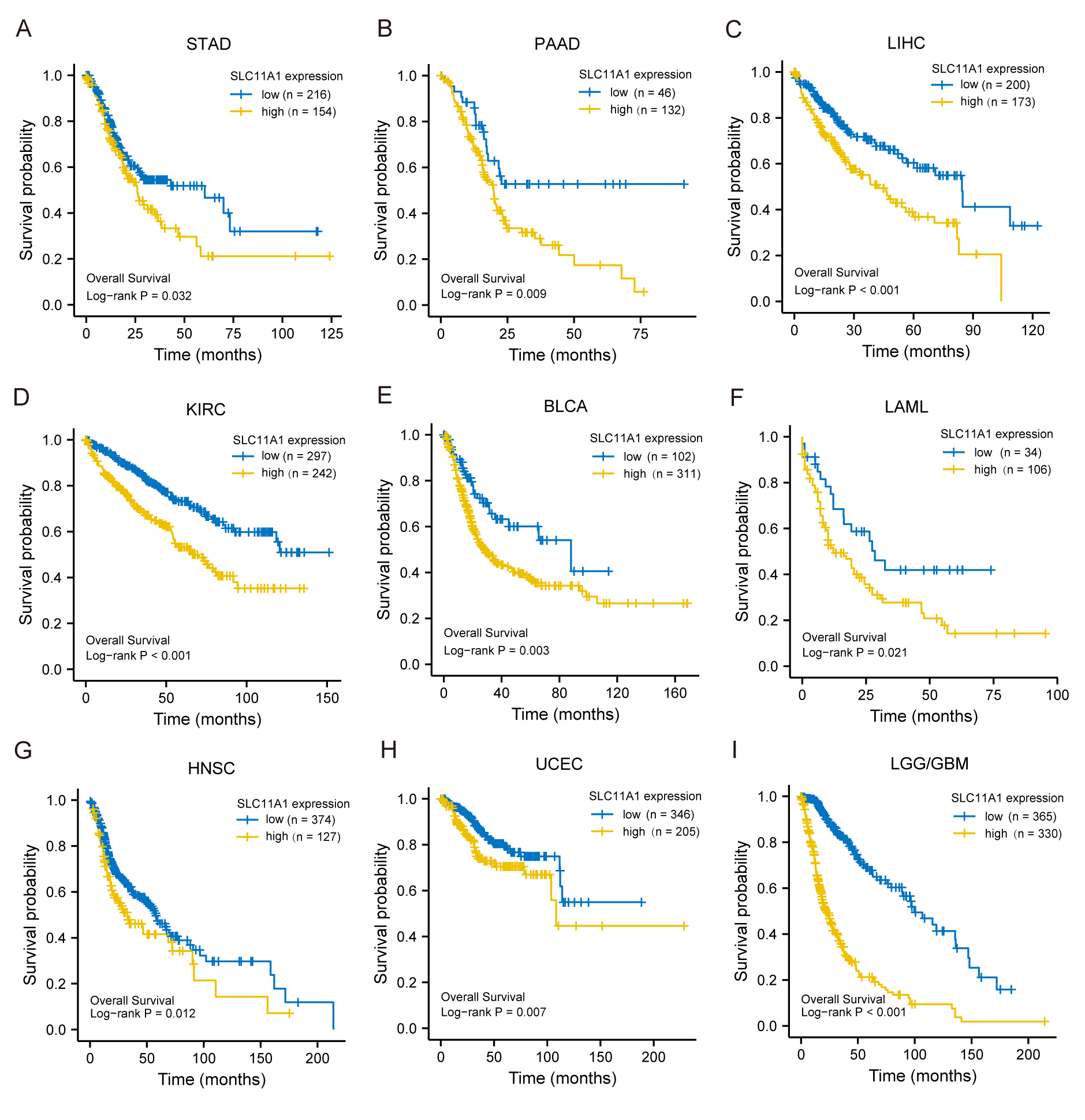
**

**Supplementary Figure 2** | Prognostic value of SLC11A1 for other digestive system tumors. (A-C) In STAD, PAAD and LIHC, high expression of SLC11A1 was associated with poor outcomes of OS. (D-I) In KIRC, BLCA, LAML, HNSC, UCEC and LGG/GBM patients, high-SLC11A1 groups had lower survival rates than low-SLC11A1 groups.
